# Atheroma of the Coronary Arteries and Its Relations to Certain Cases of Cardiac Failure

**Published:** 1885-07

**Authors:** 


### ATHEROMA OF THE CORONARY ARTERIES AND ITS RELATIONS TO CERTAIN CASES OF CARDIAC FAILURE.

At the annual meeting of the Minnesota State Medical Society, held in St. Paul, June Eighteenth and Nineteenth, DR. ROBERT H. BABCOCK, of Chicago, addressed the members upon ATHEROMA OF THE CORONARY ARTERIES AND ITS RELATION TO CERTAIN CASES OF CARDIAC FAILURE. As the subject is an interesting one, we give an abstract of DR. BABCOCK'S remarks.

He disclaimed any expectation of presenting original or novel ideas, hoping only to call attention to a subject which appeared to him to possess great practical interest, yet was apt to be overlooked by the general practitioner in the press of more obvious matters. It was the subject of heart weakness (heart decay) in persons past middle age, in consequence of degeneration of the cardiac muscle resulting from atheroma of the coronary or nutrient arteries of the heart. The picture is a common one, though presented under varying aspects. The speaker then described briefly three classes or types of cases, which he considered illustrative of the disease. First, a strong man of about sixty years of age, complains of gradual loss of strength for a few months past and of slight breathlessness on exertion, possibly also of vague pain in the region of the heart : that is all. He has never been seriously ill and the physician fails to discover anything that proves satisfactory or that definitely implicates the heart. The pulse exhibits no marked deviation from normal and the cardiac impulse is in its right position, only perhaps a trifle weak. The heart sounds are free from murmur, the first being perhaps slightly less booming than natural and the second somewhat more pronounced. At a loss for more satis factory explanation of his patient's condition, the physician says he is only a little overworked, prescribes rest and tonics, and thinks he will improve. To his great surprise the man a few months subsequently falls unconscious and with deeply congested face and neck quickly expires; or perhaps in an unusually severe attack of angina pectoris his heart ceases to beat. It is pronounced cerebral or pulmonary apoplexy. It is however sudden cardiac failure.

In another class of cases, the physician is called to see a man past middle age, who upon unusual exertion was seized with great dyspnœa and is now panting for breath. The heart's action is feeble, pulse somewhat irregular, and there is more or less bronchitis; praecordial dullness may or may not be increased. The man has evidently over-exerted himself and is now suffering with asthma, the doctor thinks. A hopeful view of his case is entertained and remedies are prescribed. But, instead of improving, the patient goes on from bad to worse, and finally after months of distress from dyspnœa, dropsy, angina, etc., death mercifully terminates the struggle.

The third class is exemplified by a person of advanced age who has for years been unequal to much exertion by reason of weakness, breathlessness, vague, substernal pain and dizziness. The phyician has no great difficulty in recognizing heart weakness, but attributes it to a general decay of vitality and and impaired digestion.

The real explanation of these and similar cases, DR. BABCOCK thought to be degeneration of the heart muscle due to atheroma or sclerosis of the coronary arteries. He then briefly described the pathology of atheroma and enumerated its causes as recognized by English and German observers, the chief being cardio-vascular strain and old age. This arterial degeneration is observed to affect most frequently the aorta and its large branches, the cerebral, coronary and splenic arteries and those of the extremities. It is not unusual to discover after death, atheroma of the aorta and coronary arteries when it had not invaded the peripheral vessel sufficiently to have been recognized during life. The changes in the walls of the heart have been recently described by PROFESSOR LEYDEN. of Berlin, and the speaker derived what he had to say on this part of his subject from LEYDENS's admirable paper. In the first group, LEYDEN places those cases where the atheromatous process has not been marked and the heart has materially suffered. In the second, there are discovered areas of acute softening due to thrombi or hemorrhagic infarcts. This condition often leads to rupture of the heart. Thirdly, there are areas of fibroid degeneration in the walls of the ventricles, particularly at the apex. In consequence of the thinning produced, aneurism (of the heart) not infrequently results. In the fourth group, there is a combination of the second and third. The clinical history of these cases is different, being either acute, subacute or chronic. LEYDEN considers this degenerative occlusion of the coronary arteries as the most frequent cause of angina pectoris.

DR. BABCOCK next spoke of the diagnosis. He regards it as not always easy or definite, particularly if the radial or other peripheral arteries be not discovered as atheromatous by the touch. Yet, a careful consideration of the history, symptoms and character of the heart sounds will often lead to a correct diagnosis. If the first sound over the left ventricle be more or less toneless in contrast to the second, which particlarly in the aortic area, is sharp and ringing, and if the impulse be feeble and diffused, there is good reason to suspect degenerative change in the walls of the heart, although praecordial dullness can not be found increased.

DR. BABCOCK thought the treatment suggested itself, since it is highly rational. It consists pre-eminently of rest and every means available for improvement in the nutrition of the heart. Arsenic and strychnia are admirable heart tonics, and the former, if pushed to a point just short of producing its physiological effects, is often found to lessen the frequency and severity of the angina. As remedies efficient for the relief of the pain during the attack the speaker recommended nitrite of amyl and a one per cent. solution of nitro-glycerine. Digitalis is invaluable for the purpose of slowing, steadying and strengthening the heart's action when dilation has gained the ascendency and the pulse is irregular and rapid. In carrying off dropsy due to cardiac failure digitalis combined with squills is most effective. The doctor also discussed other remedies, as well as dwelt upon the those mentioned at greater length than has been done in this article.

Unfortunately the society did not participate in any formal discussion of the address.

